# Training Staff Across the Veterans Affairs Health Care System to Use Mobile Mental Health Apps: A National Quality Improvement Project

**DOI:** 10.2196/41773

**Published:** 2023-01-12

**Authors:** Pearl McGee-Vincent, Margaret-Anne Mackintosh, Andrea L Jamison, Katherine Juhasz, Colleen Becket-Davenport, Jeane Bosch, Timothy J Avery, Lauren Glamb, Shilpa Hampole

**Affiliations:** 1 Dissemination and Training Division National Center for PTSD Veterans Affairs Palo Alto Health Care System Menlo Park, CA United States; 2 Office of Mental Health and Suicide Prevention Veterans Health Administration Menlo Park, CA United States; 3 National Training Division Education Service Veterans Benefits Administration Washington, DC United States; 4 Department of Psychiatry Weill Cornell Medical College New York, NY United States; 5 Peninsula Vet Center Readjustment Counselling Services US Department of Veterans Affairs Menlo Park, CA United States; 6 Veterans Affairs Pacific Islands Health Care System Honolulu, HI United States

**Keywords:** mental health, mobile apps, digital health, technology, veterans, training

## Abstract

**Background:**

The National Center for PTSD, within the Department of Veterans Affairs (VA), has developed a suite of free, publicly available, evidence-informed apps that can reach an increasing number of veterans and bridge gaps in care by providing resources to those who are not engaged in mental health treatment. To expand the reach of these apps, staff across VA service lines learned about these apps, their features and limitations, and how to introduce them to veterans.

**Objective:**

This study aimed to develop, disseminate, and evaluate a training for multidisciplinary staff as part of a national quality improvement project to increase the reach of mobile mental health apps as a resource for veterans.

**Methods:**

Sites from all of VA’s 18 geographic regions enrolled in this project. At each site, a minimum of 25 VA staff members who had direct contact with veterans, including staff from the mental health service line and all other service lines, were recruited to participate. Training included a 3-hour multidisciplinary *core* module, and a 1-hour *clinical integration* module designed specifically for mental health clinicians. Owing to the COVID-19 pandemic, the trainings were adapted to a live, web-based format. Pre- and posttraining surveys assessed program reach (ie, participants enrolled per site), satisfaction, and effectiveness of the training as measured by changes in knowledge, basic skills, and behavioral intentions to use apps with veterans.

**Results:**

A total of 1110 participants representing 34 disciplines at 19 VA sites completed the training. Overall, 67% (743/1109) of participants were mental health staff members. Sites averaged 58.4 participants (SD 36.49, median [IQR] 51). Most (961/1024, 93.85%) participants were satisfied with the training and reported that they (941/1018, 92.44%) would recommend it to others. App knowledge scores significantly increased from pretraining (mean 80.8% correct, SD 15.77%) to posttraining (mean 91.1% correct, SD 9.57%; *P*<.001). At posttraining, participants also reported greater confidence in their ability to show veterans how to *download* (*z*=−13.86; *P*<.001) and *use* VA mental health apps (*z*=−15.13; *P*<.001). There was near universal endorsement by staff for their intentions to recommend apps to veterans as well as their ability to think of at least one specific veteran to whom they could recommend an app. Staff also reported a strong motivation to encourage other VA staff to share apps with veterans.

**Conclusions:**

The training far exceeded the initial goals for staff recruitment and training for all three metrics. Overall, 33% (366/1109) of participants came from service lines outside of mental health, indicating the feasibility of introducing these mental health resources during medical appointments and in other contexts.

## Introduction

To expand access to mental health self-management resources, the Department of Veterans Affairs’ (VA) National Center for PTSD develops free mental health apps (VA MH apps) that provide psychoeducation, symptom tracking, and coping tools for PTSD and other related mental health concerns [1,2]. Although these self-care apps (eg, PTSD Coach, Insomnia Coach, and Mindfulness Coach) do not replace treatment, they have the potential to bridge gaps in care by making mental health resources, including information for accessing professional care, available to veterans who experience barriers to treatment [3]. In addition, for veterans participating in evidence-based psychotherapy, treatment companion apps (eg, PE Coach for prolonged exposure therapy) can facilitate aspects of treatment protocols (eg, access to educational materials, between-session homework, and session recordings) [4].

With an estimated 10,000 mental health apps available [5] and minimal quality control or gatekeeping in the app marketplaces [6], it is important that VA staff recommend apps that are evidence-informed and do not compromise patient privacy. VA MH apps are free to download, do not require an account or log-in, do not collect or store identifiable data, and are developed with subject matter expertise [2]. Furthermore, VA MH apps are required to comply with Section 508 of the Rehabilitation Act [7], meaning that they are accessible to individuals with disabilities. Although VA MH apps are available to any member of the public, content is designed to be acceptable to a veteran user base. In fact, VA MH apps have been well received by VA patients and staff [8] and are feasible to use [9-11]. Although not designed to constitute stand-alone treatment interventions, VA MH apps contain active components of evidence-based psychotherapies [12]. Naturalistic use data indicate that the PTSD Coach app, in particular, has promising potential as a public health resource [13,14]. In addition, PTSD Coach users who received clinician support when using the app in primary care were more likely to accept a mental health referral and attend PTSD treatment [15].

Despite the potential benefits of VA MH apps and the increasing number of veterans with access to a smartphone or tablet, a minority of veterans engaged in VA care have heard of or used these apps [16]. To increase awareness and use of a similar suite of apps, Armstrong et al [17] implemented a training program over a 3-year period (2014-2017) in which 760 mental health clinicians, primarily in the Department of Defense (DoD), were trained on how to integrate VA and DoD apps into mental health treatment. Given that many VA patients with mental health needs do not engage in formal mental health treatment [18], training staff outside mental health settings to use these apps could be an important way to reach such patients. As VA MH apps for self-care can be introduced by any VA staff member (eg, chaplains, primary care physicians, and peer support specialists), a training program was developed to meet the learning needs of staff who are part of the mental health service line (MHSL) as well as all other service lines (AOSLs). The training program was designed to increase participants’ (1) knowledge about VA MH apps, (2) confidence in their ability to download and use these apps with veterans, and (3) intention to use VA MH apps with veterans in the months following the training. Originally designed to be delivered in-person, the training was modified to a live, web-based format due to the COVID-19 pandemic and the resulting travel restrictions.

This training was part of a national quality improvement project to increase veterans’ access to VA MH apps as a resource. As training is important but often insufficient to implement new practices [19], the project team used Implementation Facilitation [20] to support the implementation and sustainment of VA MH apps as an integrated part of care at participating sites. Implementation Facilitation is a widely used set of practices that leverages trained Facilitators along with tailored organizational strategies to help health care organizations overcome barriers and increase the adoption of evidence-based or promising practice [20]. This paper will focus primarily on the evaluation of the training, and a subsequent manuscript will elaborate on the results of the implementation activities that followed.

## Methods

### Formative Evaluation

Formative evaluation (FE) is a process of evaluating and modifying the content and design of training as it is developed [21]. As part of the FE, interviews were conducted with VA staff to identify learner needs, tailor training objectives, and adapt content from existing training materials. For example, FE helped the project team to determine how to modify the training content to accommodate the needs of staff from different disciplines. This process was ongoing, meaning that the training was modified over time, as new information was collected from participants. This information was obtained formally (via surveys) and informally (via verbal participant feedback), as well as through interviews with Facilitators and members of the project team who delivered the training and served as the primary point of contact with participating sites.

### Recruitment

#### Site-Level

To maximize the reach of the project, sites from each of VA’s 18 geographic regions that span the entire United States were invited to apply with the goal of enrolling one participating site per region. These sites were identified, via FE interviews, as having a site champion (defined below), leadership buy-in, and interest in participation from mental health, and at least one other service line.

Sites interested in enrolling were required to (1) identify a site champion, or “mHealth Specialist,” to lead the initiative locally, including coordinating with Facilitators for training and subsequent implementation activities; (2) identify at least 25 staff participants; (3) provide time for enrolled staff to participate in a live 4-hour training; (4) identify and engage key stakeholders (eg, managers and leaders from across programs) to participate in a site visit to include leadership briefings and an implementation planning session; and (5) agree to participate in the 3-month implementation initiative following the training.

#### Participant-Level

All staff members at enrolled sites who had direct contact with veterans were eligible to participate, pending supervisor approval. In addition to those who provide direct clinical care, other staff members such as medical support assistants, librarians, and peer support specialists were also eligible. Facilitators provided tailored support and recruitment materials (eg, information packets and flyers) to help sites identify and recruit staff to participate in the project. Ultimately, the sites used several recruitment strategies. For example, mHealth Specialists and service-line leadership at most sites disseminated a project recruitment flyer via email with an electronic enrollment link to all service line staff. Other sites worked with a public affairs officer to email the flyer to all staff. At most sites, the participants were self-selected. However, in some instances, supervisors from individual teams nominated representatives to participate in the training. Occasionally, the entire team or service line was directed by local leadership to enroll in the project.

### Design of the VA MH Apps Training

#### Development of the Training

The training curriculum was adapted from training by Armstrong et al [17] and trainings developed previously by the project team. These include conference workshops on VA MH apps [22], a web-based course [23], and a book chapter describing how to integrate apps into care [24]. These materials were integrated with information collected during FE interviews and adapted to be relevant for both MHSL and AOSL participants with varying levels of skills and experience using the VA MH apps.

The project team created a 3-hour *core* training for all staff, regardless of discipline, and a 1-hour *clinical integration* module designed specifically for staff who provide mental health treatment. Topics for the core training included the rationale for recommending VA MH apps for self-care, live demonstrations of self-care apps, practical suggestions for introducing apps to veterans, and an overview of related resources for veterans and VA staff. The live app demonstrations featured the 2 most downloaded VA MH apps: PTSD Coach and Mindfulness Coach. In addition, the digital Safety Plan, a newly added feature of the PTSD Coach app, was designed to provide a readily accessible, mobile option for the safety planning for suicide intervention worksheet. The training provided practical examples for introducing apps in a range of settings and scenarios to help participants determine how to best fit these resources into their workflows. The 1-hour clinical integration module then focused on apps as an adjunct to mental health treatment. Topics relevant to clinicians (eg, education on obtaining informed consent when integrating apps into care and demonstration of treatment companion apps) were covered in this section. All training sessions were delivered by Facilitators.

Consistent with FE, the project team continued to refine the training based on systematic documentation of feedback, including participant input during training, feedback on posttraining surveys, and posttraining interviews with Facilitators. Some examples of changes made during the course of the project included the addition of a live demonstration of the Anger & Irritability Management Skills (AIMS) app during the clinical integration hour, rearranging material to improve flow, and the creation of flexible breakout groups in the clinical integration module, in which participants could choose the treatment companion app they would like to see (eg, PE Coach vs CPT Coach). Training modifications were often larger in scope following the first few training sessions; however, small modifications were made as required through the course of the training period.

In addition to increasing participants’ awareness of VA MH apps and their ability to download and use them competently with veterans in accordance with their scope of practice, the third objective of the training was to increase participants’ intentions to apply what they had learned. As part of the training, participants were encouraged to share VA MH apps with veterans and other staff over the course of the 3 months following the training and beyond.

#### Site Visits

The trainings were designed to take place during an in-person site visit led by 2 Facilitators at each participating site. Sites elected to offer 1 or 2 staff training days, depending on the number of participants enrolled, the capacity of the conference room (for in-person visits), and staff schedules. The participants completed the surveys (described below) before and after each training. In addition to the trainings, site visits included an implementation planning session and leadership briefings, which were designed to support the application of training and the sustainment of new practices.

#### Adaptations Due to the COVID-19 Pandemic

Site visits, including live training sessions, were scheduled to take place on a rolling basis over 12 months, from January to December 2020, at a rate of approximately 3 visits every 2 months. The first 3 site visits occurred in person before the COVID-19 pandemic. Several in-person site visits scheduled for the spring were postponed and ultimately converted to a live, web-based format. After a pause and recalibration, the first web-based site visit occurred in June 2020, and the remaining 15 site visits occurred via web from July to December.

Instead of each visit spanning 1 to 3 days on-site, web-based site visit activities could be spread out over 1 to 2 weeks to accommodate multiple schedules. Trainings were also adapted to make them more engaging in web-based formats [25,26]. For example, breakout groups, multimedia elements, and polls were added to foster audience participation. Facilitators encouraged participants to interact in the chat by offering prompts and answering questions in real time. The breakout groups discussed pertinent training topics and promoted the participants’ engagement. Finally, Facilitators included an interactive exercise after each live demonstration of an app, which allowed participants to gain hands-on experience navigating through the app to find answers to prompts. QR codes were also added to the training slides and other project materials so that participants could easily access them during the web-based training.

### Measures

#### Survey of Participant Characteristics, Attitudes, and Use of Technology

Before training, participants completed web-based surveys regarding their demographics and role within VA, as well as their attitudes toward, skills, comfort with, and use of VA MH apps. Items measuring basic skills, as well as knowledge about and behavioral intentions to use VA MH apps, were assessed again immediately posttraining. Individual items from these sources were used to describe the sample and compare the background characteristics between MHSL and AOSL staff. Participants who reported serving both the MHSL and AOSL were included in the MHSL group.

#### Training Program Reach

To assess the reach of the training, the metrics used were (1) a minimum of 25 participants per site and (2) participation from both MHSL and AOSL staff.

#### Training Satisfaction

Two items in the posttraining survey were used to assess the participants’ satisfaction with the program. The first item asked how satisfied each person was with the training and the second evaluated whether they would recommend the training to others. Each item was rated on a 5-point scale ranging from *strongly agree* to *strongly disagree* with a midpoint of *neither agree nor disagree*. We reported the percentage of participants who either *strongly agreed* or *agreed* with the 2 questions as our measure of program satisfaction.

#### Training Effectiveness

Three domains were used to assess the effectiveness of the training program: (1) increase in knowledge about VA MH apps, (2) acquisition of the basic skills needed to demonstrate how to *download* and *use* VA MH apps with veterans, and (3) participants’ behavioral intentions to use VA MH apps with veterans and other staff in the 3 months following training.

##### Knowledge Check

Knowledge about using VA MH apps was assessed using a 16-item knowledge test created specifically for this project. The item content was developed by Facilitators and other subject matter experts and was based on the information highlighted during the training. The percentage of correctly answered items was used as a knowledge metric.

##### Basic Skills

Possession of the basic skills to use VA MH apps with veterans was measured using 2 items assessing confidence in one’s “knowledge and skills necessary to demonstrate to Veterans how to *download* VA apps” and “knowledge and skills necessary to demonstrate to Veterans how to *use* VA apps.” Each item was rated on a 5-point scale ranging from *strongly agree* to *strongly disagree* with *neither agree nor disagree* as the midpoint.

##### Behavioral Intentions

Behavioral intentions to use VA MH apps with veterans were measured using 3 items adapted from Kim and Park, 2012 [27], which are grounded in the Theory of Planned Behavior by Ajzen [28]. The questions were (1) “I will recommend VA apps to Veterans in the next 3 months,” (2) “I can think of at least one Veteran I plan to recommend or use a VA app within the next month,” and (3) “I will encourage other VA staff to recommend or use VA apps with Veterans.” Each item was scored on a 4-point scale: *definitely will, probably will, probably will not,* and *definitely will not*.

### Statistical Analysis

Analyses focused on descriptive statistics detailing the composition and characteristics of the key groups and primary outcomes. Inferential statistics, including 2-sided *t* tests and general linear models, were used to analyze continuous outcomes. Chi-square tests were used to compare independent groups, and Wilcoxon signed-rank tests were used to compare paired groups when analyses included nominal and ordinal outcomes. In addition to assessing the statistical significance of the results, we provided effect size estimates. For *t* tests, standardized mean differences (Cohen *d*) are presented using the conventional interpretative guidelines of 0.20=small, 0.50=medium, and 0.80=large effects [29]. Effect sizes for the general linear models are summarized using partial *η*^2^ with interpretative guidelines for 0.01=small, 0.09=medium, and 0.25=large effects [29]. All the available data were used for each analysis. Because multiple statistical tests were conducted, family-wise error was controlled using the Holm step-down method, which yielded a criterion of .001 for unadjusted *P* values [30]. We report unadjusted *P* values, and those equal to or smaller than this criterion (*P*=.001) were considered statistically significant. All analyses were conducted using SPSS (version 24.0; IBM Corp).

Missing data rates varied from less than 1% for many of the background variables (eg, service lines and disciplines), approximately 25% (about 278/1110) for basic skills and behavioral intention items, and approximately 30% (about 333/1110) for sociodemographic items (eg, age, gender, and education), which appeared at the end of the survey. Of the 1110 participants enrolled in the project, 862 (77.66%) started the pretraining survey and 1023 (92.16%) started the posttraining survey. As most statistical procedures used (ie, chi-square analyses and Wilcoxon rank sum tests) do not have easily accessible routines to pool the results required to use modern multiple imputation procedures, sensitivity analyses were conducted using 20 multiply imputed data sets, in which (1) the analyses described were replicated using the 20 imputed sets, and (2) analyses using similar but more complex statistical procedures answering similar questions (eg, binary or ordinal regression) were explored. The patterns of the results remained unchanged across the analyses. Thus, initial analyses are provided.

### Ethics Approval

Ethics approval was obtained from the Stanford Institutional Review Board (Protocol #60207), and this project was determined not to qualify as human subjects research.

## Results

### Sample Description

The sample consisted of 1110 participants and included 42.42% (467/1101) staff members who reported that they provided mental health services or psychotherapy. In addition, 53.18% (569/1070) of participants reported conducting safety planning interventions for suicide prevention. Tables 1 and 2 present the staff work characteristics and sociodemographic information of MHSL staff (743/1109, 67%) and AOSL staff (366/1109, 33%, one participant’s service line could not be identified). A total of 39 participants reported serving both on the mental health and another service line and were counted in the MHSL group. The 2 staff types were similar in gender, disability status, years of experience working with veterans, and average number of hours per week spent interacting with veterans. The 2 groups differed on two background characteristics. First, MHSL staff members were slightly younger (mean 44.2, SD 10.16) compared with AOSL staff members (mean 46.0, SD 10.9), t_743_=2.11, *P*=.04, Cohen *d*=0.17. Second, MHSL staff reported higher educational attainment compared with AOSL staff, *χ*^2^_5_=73.41, *P*<.001.

The 1110 participants represented a range of service lines with 67% (743/1109) from MHSL, 14.92% (165/1106) from primary care, 14.29% (158/1106) from Medical Specialty service lines, and 7.96% (88/1106) from other service lines. Across service lines, 68.17% (754/1106) of the participants worked in outpatient programs, 20.80% (230/1106) reported working across 2 or more program types, 9.13% (101/1106) worked in inpatient or residential programs, and 1.90% (21/1106) reported working in research or administrative roles. The participants represented 34 different disciplines (Table 3).

**Table 1:** Participants’ age and work characteristics (N=862).

Characteristics	MHSL^a^ staff (n=602^b^)			AOSL^c^ staff (n=260^b^)			Statistical results		
	n	mean (SD)	n		mean (SD)	*t* test (*df*)		*P* value	Cohen *d*^d^
Age (years)	521	44.2 (10.2)	234		46.0 (10.9)	2.1 (753.0)		.04	0.2
Experience working with veterans (years)	549	8.4 (5.6)	243		8.0 (6.4)	−0.87 (413.5)		.41	−0.1
Hours interacting with veterans per week	545	24.2 (11.0)	239		23.5 (13.8)	−0.76 (376.6)		.49	−0.1

^a^MHSL: mental health service line.

^b^862 of the 1110 participants (77.66%) enrolled in the project responded to the pretraining survey.

^c^AOSL: all other service lines.

^d^Standardized mean difference representing between-group effect size as Cohen *d*, guideline for effect sizes: small=0.20, medium=0.50, and large=0.80.

**Table 2:** Participants’ sociodemographic descriptors (N=862).

Characteristics		MHSL^a^ staff (n=602^b^), n (%)	AOSL^c^ staff (n=260^b^), n (%)	Statistical results			
				*χ*^2^ (*df*)		*P* value	
**Gender**					7.4 (3)		.06
	Woman	397 (71.8)	193 (79.1)				
	Man	144 (26)	46 (18.9)				
	Nonbinary	0 (0)	1 (0.4)				
	Prefer not to say	12 (2.2)	4 (1.6)				
**Race or ethnicity^d^**							
	American Indian or Alaska Native	11 (1.3)	3 (0.3)	0.5 (1)		.47	
	Asian or Asian American	37 (4.3)	23 (2.7)	2.0 (1)		.16	
	Black or African American	75 (8.7)	43 (5)	2.5 (1)		.11	
	Hispanic, Latino, or Spanish	50 (5.8)	20 (2.3)	0.1 (1)		.76	
	Native Hawaiian or other Pacific Islander	5 (0.8)	4 (0.5)	0.9 (1)		.35	
	White	408 (47.4)	165 (19.2)	1.6 (1)		.21	
	Another race or ethnicity	6 (0.7)	6 (0.7)	2.3 (1)		.13	
**Education**					73.4 (5)		<.001
	High school or General Educational Development	6 (1.1)	2 (0.8)				
	Some college or Associate’s Degree	35 (6.3)	57 (23.9)				
	Bachelor’s Degree	47 (8.5)	34 (14.4)				
	Some graduate school	11 (2)	13 (5.5)				
	Master’s degree	245 (44.4)	79 (33.1)				
	Doctorate, medical degree, or equivalent	208 (37.7)	53 (22.3)				
**Disabilities reported^e^**					1.8 (2)		.40
	None	483 (86.3)	205 (83.7)				
	1	65 (11.6)	31 (12.7)				
	≥2 (2-5)	12 (2.1)	9 (3.7)				

^a^MHSL: mental health service line.

^b^862 of the 1110 participants (77.7%) enrolled in the project responded to the pretraining survey.

^c^AOSL: all other service lines.

^d^Participants could endorse multiple races or ethnicities. Therefore, race and ethnicity were analyzed separately.

^e^Self-reported disabilities included hearing impairment (33/805, 4.1%), visual impairment (31/805, 3.9%), mobility impairment (17/805, 2.1%), learning disability (12/805, 1.5%), and any other disability or impairment (58/805, 7.2%). Overall, 59/805 (7.3%) stated that they preferred not to report their disability status.

**Table 3:** Participants’ disciplines (N=1107).

Disciplines	Values, n (%)
Social Workers	256 (23.1)
Nurses	240 (21.7)
Psychologists	232 (21.0)
Peer Support Specialists	61 (5.5)
Chaplains	41 (3.7)
Medical Support Assistants	40 (3.6)
Psychiatrists	39 (3.5)
Medical Doctors	33 (3)
Audiologists	31 (2.8)
Other disciplines^a^	134 (12.1)

^a^Other disciplines (reported as n out of 1107): Vocational Rehabilitation Specialist (19/1107, 1.72%), Pharmacists (15/1107, 1.36%), Licensed MH Counselor (12/1107, 1.08%), Psychology Technicians (9/1107, 0.81%), Physician Assistants (8/1107, 0.72%), Administrative Personnel (7/1107, 0.63%), Recreational Therapist (7/1107, 0.63%), Program Support Specialists (6/1107, 0.54%), Communication Specialists (5/1147, 0.45%), Dietitians (5/1107, 0.45%), Occupational Therapists (5/1107, 0.45%), Patient Advocates (5/1107, 0.45%), Coaches (4/1107, 0.4%), Medical Technicians (4/1107, 0.4%), Physical Therapists (4/1107, 0.36%), Addiction Therapists (3/1107, 0.27%), Librarians (3/1107, 0.27%), Volunteers (3/1107, 0.27%), Music Therapists (2/1107, 0.18%), Program Coordinators (2/1107, 0.18%), Learning Resource/Education Staff (2/1107, 0.18%), Dental Assistant (1/1107, 0.09%), Outreach Specialist (1/1107, 0.09%), Police Officer (1/1107, 0.09%), and Research Assistant (1/1107, 0.09%).

### Technology-Related Participant Characteristics

Table 4 summarizes the participants’ ownership, attitudes, and skills using VA MH apps broken down by staff type at baseline. Both groups reported a high degree of personal smartphone ownership (828/862, 96.06%). The vast majority (753/830, 90.72%) were either *very comfortable* or *quite comfortable* using apps, with most spending at least 1 hour per day using them (719/844, 85.19%). Most participants reported being confident in their skills to show veterans how to *download* apps (655/841, 77.88%) and showing veterans how to *use* VA MH apps (593/839, 70.68%) at baseline.

The 2 staff types differed in 3 ways related to technology. First, MHSL staff (204/602, 33.89%) were more likely than AOSL staff (51/260 19.62%) to have government-issued smartphones, *χ*^2^_1_=17.75, *P*<.001. Second, MHSL staff had higher mean percentage correct scores on the knowledge check at pretraining (mean 82.1% correct, SD 16%) compared with the AOSL staff, who averaged 76.5% correct (SD 15.22%), t_829_=−4.74, *P*<.001, Cohen *d*=−0.36. Finally, MHSL staff (550/574, 95.82%) were more likely to report that they *definitely* or *probably* could think of at least one veteran who they planned to use apps with compared with AOSL staff (215/252, 85.32%), *χ*^2^_2_=29.55, *P*<.001.

**Table 4:** Participants’ technological skills and intentions for Veterans Affairs mental health (VA MH) apps use at baseline (N=862).

					MHSL^a^ staff (n=602^b^), n (%)		AOSL^c^ staff (n=260^b^), n (%)		Statistical results				
									*χ*^2^ (*df*)			*P* value	
**Owns a personal smartphone**										0.7 (1)			.39
	Yes				576 (95.7)		252 (96.9)						
	No				26 (4.3)		8 (3.1)						
**Has a government-issued smartphone**										17.8 (1)			<.001
	Yes				204 (33.9)		51 (19.6)						
	No				398 (66.1)		209 (80.4)						
**Comfort using mobile devices**										2.9 (2)			.24
	Not at all or A little comfortable				45 (8.1)		30 (11.9)						
	Quite comfortable				204 (35.4)		87 (34.4)						
	Very comfortable				326 (56.5)		136 (53.8)						
**Hour per day spent using apps**										6.0 (4)			.20
	I do not use apps	13 (2.2)				4 (1.6)							
	<1 hour per day	76 (12.9)				32 (12.5)							
	1-2 hour per day	238 (40.5)				84 (32.8)							
	3-4 hour per day	168 (28.6)				88 (34.4)							
	>4 hour per day	93 (15.8)				48 (18.7)							
**Skills to demonstrate how to *download* apps**										0.8 (4)			.93
	Strongly agree			204 (34.9)			83 (32.3)						
	Agree			251 (43)			117 (45.5)						
	Neither Agree nor Disagree			71 (12.2)			30 (11.7)						
	Disagree			49 (8.4)			22 (8.6)						
	Strongly disagree			9 (1.5)			5 (1.9)						
**Skills to demonstrate how to *use* apps**										1.5 (4)			.83
	Strongly agree		162 (27.8)				69 (27)						
	Agree		245 (42.1)				117 (45.7)						
	Neither Agree or Disagree		95 (16.3)				36 (14.1)						
	Disagree				72 (12.3)		29 (11.3)						
	Strongly disagree				9 (1.5)		5 (2)						
**Recommend VA MH apps to veterans (BI^d^ item 1)**										11.1 (2)			.004
	Definitely will				356 (61.7)		143 (56.3)						
	Probably will				211 (36.6)		96 (37.8)						
	Probably will not or Definitely will not				10 (1.7)		15 (5.9)						
**Think of at least 1 Veteran I plan to use apps with (BI item 2)**										29.6 (2)			<.001
	Definitely will				335 (58.4)		121 (48)						
	Probably will				215 (37.5)		94 (37.3)						
	Probably will not or Definitely will not				24 (4.2)		37 (14.7)						
**Encourage other VA staff to use apps (BI** **item 3)**										1.1 (2)			.58
	Definitely will				307 (53.1)		142 (55.9)						
	Probably will				237 (41)		101 (39.8)						
	Probably will not or Definitely will not				34 (5.9)		11 (4.3)						

^a^MHSL: mental health service line.

^b^Of the 1110 participants enrolled in the project, 862 (77.66%) responded to the pretraining survey.

^c^AOSL: all other service lines.

^d^BI: behavioral intentions.

### Training Program Reach

The program exceeded the goals set for participant recruitment. Across the 19 sites, 1110 staff attended the training program with an average of 58.4 participants per site (SD 36.49, median [IQR] 51). This was 247% of the minimum goal of 25 participants from 18 locations. All sites met the goal of recruiting both MHSL and AOSL staff members to participate in the program.

### Training Satisfaction

Table 5 summarizes the data from the posttraining survey, which included measures of training satisfaction. There were no significant differences based on staff type for training satisfaction, *χ*^2^_3_=7.93, *P*=.047 or for willingness to recommend training to others, *χ*^2^_3_=7.71, *P*=.052. The vast majority (961/1024, 93.85%) of participants were satisfied with the training and 92.44% (941/1018) would recommend the training to others.

**Table 5:** Participants' training satisfaction, basic technology skills, and intentions for Veterans Affairs mental health (VA MH) apps use from the posttraining survey (N=1023).

			MHSL^a^ staff (n=686^b^), n (%)		AOSL^c^ staff (n=337^b^) , n (%)		Statistical results				
							*χ*^2^ (*df*)			*P* value	
**Satisfaction with training**								7.9 (3)			.047
	Strongly agree	393 (57.3)		203 (60.1)							
	Agree	241 (35.1)		124 (36.7)							
	Neither agree nor disagree	35 (5.1)		9 (2.7)							
	Disagree or strongly disagree	17 (2.5)		2 (0.6)							
**Would recommend training**								7.7 (3)			.052
	Strongly agree	421 (61.5)		206 (61.7)							
	Agree	201 (29.4)		113 (33.8)							
	Neither agree nor disagree	43 (6.3)		11 (3.3)							
	Disagree or strongly disagree	19 (2.8)		4 (1.2)							
**Skills to demonstrate how to *download* apps**								3.0 (4)			.55
	Strongly agree	384 (56.2)		182 (54.3)							
	Agree	278 (40.7)		136 (40.6)							
	Neither agree or disagree	13 (1.9)		12 (3.6)							
	Disagree	4 (0.6)		3 (0.9)							
	Strongly disagree	4 (0.6)		2 (0.6)							
**Skills to demonstrate how to *use* apps**								1.7 (4)			.80
	Strongly agree	344 (50.1)		160 (47.5)							
	A	314 (45.8)		161 (47.8)							
	Neither agree or disagree	19 (2.8)		10 (3)							
	D	4 (0.6)		4 (1.2)							
	Strongly disagree	5 (0.7)		2 (0.6)							
**Recommend apps to veterans (BI^d^ item 1)**								11.8 (2)			.003
	Definitely will	577 (84.5)		255 (75.9)							
	Probably will	96 (14.1)		76 (22.6)							
	Probably will not or Definitely will not	10 (1.5)		5 (1.5)							
**Think of at least 1 Veteran I plan to use VA MH apps with (BI item 2)**								29.3 (2)			<.001
	Definitely will	556 (81.3)		225 (67)							
	Probably will	116 (17)		92 (27.4)							
	Probably will not or Definitely will not	12 (1.7)		19 (5.6)							
**Encourage other VA staff to use VA MH apps (BI item 3)**								1.7 (2)			.43
	Definitely will	486 (71.3)		235 (70.4)							
	Probably will	178 (26.1)		94 (28.1)							
	Probably will not or Definitely will not	18 (2.6)		5 (1.5)							

^a^MHSL: mental health service line.

^b^Of the 1110 participants enrolled in the project, 1023 (92.2%) responded to the posttraining survey.

^c^AOSL: all other service lines.

^d^BI: behavioral intentions.

### Training Effectiveness

#### Knowledge Check

For knowledge check scores, a generalized linear model was used to evaluate the between-group effect for staff type (MHSL vs AOSL staff) and the repeated measures effect of time (pre- and posttraining) as well as their interaction. Controlling for family-wise error, the interaction was not statistically significant, *F*_1,758_=7.89, *P*=.005, partial *η*^2^=0.010. The main effect for staff type was statistically significant, *F*_1,773_=25.77, *P*<.001, partial *η*^2^=0.033 (small effect). Collapsing across pre- and posttraining time points, MHSL staff had significantly higher mean scores (mean 87.2% correct, SD 12.56%) compared with AOSL staff (mean 83.1% correct, SD 7.64%). The main effect for time was statistically significant. When collapsing across staff types, the mean knowledge check scores significantly increased from pretraining (mean 80.8%, SD 15.77%) to posttraining (mean 91.1% correct, SD 9.57%), *F*_1,758_=318.79, *P*<.001, *η*^2^=0.296 (large effect).

#### Basic Skills

Wilcoxon signed-rank tests were conducted to assess the pre- to posttraining changes on the 2 items that asked participants to rate their confidence in their ability to demonstrate how to *download* and *use* apps with veterans. There were statistically significant increases in participants’ confidence ratings for both how to *download* apps, *z*=−13.86, *P*<.001 and for how to *use* apps, *z*=−15.13, *P*<.001. For both items, the *strongly disagree* and *disagree* response categories were collapsed because there were only a few responses in each category. The percentage of staff who *agreed* or *strongly agreed* to having the knowledge and skills to show veterans how to *download* apps (655/841, 77.33%) and how to *use* apps (593/839, 70.68%) at baseline increased to 96.27% (980/1018) for *downloading* apps and 95.70% (979/1023) for *using* apps following training. There were no significant differences on either item based on staff type (download apps: *χ*^2^_4_=3.04, *P*=.55; use apps: *χ*^2^_4_=1.67, *P*=.80).

#### Behavioral Intentions

Wilcoxon signed-rank tests were used to compare pre- and posttraining changes for the three items measuring participants’ behavioral intentions to use mobile apps with veterans. Statistically significant increases at posttraining compared with pretraining for intentions to *use* VA MH apps with veterans were found for all 3 items (see the Methods section > Training Effectiveness > Behavioral Intentions section of this report), item 1, *z*=−11.77, *P*<.001; item 2, *z*=−11.85, *P*<.001; and item 3, *z*=−9.94, *P*<.001. Similar to the findings on the pretraining survey for the second Behavioral Intentions item, there were statistically significant differences at posttraining between MHSL and AOSL staff on the ability to identify at least one veteran with whom they plan to use apps, *χ*^2^_2_=29.32, *P*<.001 (Table 5)**.** Specifically, compared with AOSL staff (225/336, 66.96%), MHSL staff (556/684, 81.29%) were significantly more likely to report that they *definitely* could think of at least one veteran with whom they plan to use apps following training. However, looking more broadly at this item after training, nearly all staff (989/1020, 96.96%) reported that they could *definitely* or *probably* identify a veteran with whom they planned to use apps.

## Discussion

### Principal Findings

A training program was implemented to facilitate VA staff’s use of VA MH apps with veterans seen in mental health and non–mental health settings. The number of participants (N=1110) exceeded the project’s recruitment target of 25 per site, suggesting a higher-than-anticipated staff interest in this training. Participants represented 34 disciplines, and 33% (366/1109) of participants worked in settings other than mental health, reflecting the relevance of VA MH apps in diverse settings across the health care system. Given that most VA patients do not use mental health services, with only 22% of those diagnosed with a mental illness receiving psychotherapy and over half dropping out by the second session [31], the level of participation by staff outside mental health settings is promising for veterans who might otherwise not be reached. Furthermore, the training was well received: over 90% of staff members reported being satisfied with the training and said that they would recommend it to others.

In addition to the successful recruitment and engagement of participants, measures of the effectiveness of training indicated significant increases in (1) key knowledge about VA MH apps, (2) confidence in the ability to use VA MH apps with veterans, and (3) behavioral intentions to use VA MH apps with veterans. AOSL and MHSL staff knowledge was high at pretraining, particularly for MHSL staff, but increased significantly from pre- to posttraining for both groups. In terms of skills, about 96% of participants reported being comfortable showing veterans how to both download (980/1018, 96.27%) and use (979/1023, 95.70%) VA MH apps after the training. Finally, although behavioral intentions to use VA MH apps with veterans at pretraining were high, they also increased following training. Nearly all participants endorsed intentions to recommend apps to veterans and could think of at least one specific veteran to whom they could recommend an app.

To summarize, both AOSL and MHSL staff made significant gains in their knowledge and intention to use VA MH apps. Both before and directly after the training, AOSL staff had less knowledge of VA MH apps and were less likely than their MHSL counterparts to be able to think of a Veteran to whom they could introduce a VA MH app. However, the differences between staff types were small, and both groups indicated an increased likelihood of using MH apps with veterans after training.

### Limitations

This quality improvement project relied on voluntary participation of VA employees at facilities that attained leadership support for the implementation of VA MH apps. The extent to which the positive outcomes were observed could be attributed to this being a voluntary sample of early adopters. Furthermore, because we focused on VA Medical Centers, it is unclear how these findings might generalize to other health care settings. Research is needed to understand the potential impacts of introducing VA MH apps outside mental health contexts. For example, future studies could further explore whether VA MH apps help individuals self-refer to mental health services.

Next, there were concerns about ceiling effects at baseline for some key metrics (staff members’ knowledge and confidence in their skills to use VA apps). However, significantly more staff members met the goal criteria after the training. In addition, we did not deliver the same exact training across sites, as we modified our training based on feedback from each site. We also adapted to unanticipated circumstances by switching from an in-person to a live, web-based training model owing to COVID-19 restrictions. The effects of these two factors on outcomes are unclear, but they are consistent with quality improvement work.

Finally, the self-reported survey data presented in this paper were collected before and immediately after the training and did not directly address how attitudes and beliefs were translated into action. Participants reported intentions to act, which, according to the Theory of Planned Behavior [28], can inform actual behavior. This limitation will also be addressed in a subsequent manuscript that will discuss posttraining implementation efforts and, specifically, participants’ adoption of VA MH apps.

### Comparison With Prior Work

To our knowledge, this is the first VA MH app training program to demonstrate that lay health care staff are willing and able to be trained to introduce patient-facing mental health apps in a variety of VA settings. Armstrong et al [17] trained DoD and VA behavioral health providers on a similar suite of mental health applications. Although the authors used different measures of satisfaction, skill acquisition, and behavioral intentions, they similarly showed that participants rated the program well, had skill improvements, and intended to adopt practices following training. The results were promising: most of the 760 clinicians trained over 3 years demonstrated knowledge gains and reported immediately following the training that they believed the training was helpful and intended to apply what they learned in their clinical work. The current project adds to the extant literature by including nonmental health staff and training more staff in a shorter timeframe (1 year vs 3 years) while also increasing staff’s knowledge and behavioral intentions. Importantly, this project coincided with a global pandemic and a concurrent increase in telehealth [32], which may have generated increased interest in digital health technology, such as mental health apps, that can be used remotely.

### Conclusions

The VA MH apps training far exceeded the initial goals for staff recruitment and training in all three metrics: program reach, participant satisfaction, and training effectiveness. Over 33% (366/1109) of the participants came from AOSLs, which suggests the utility of VA MH apps across the health care system. Veterans’ access to mental health resources is not limited to traditional referrals to mental health providers. The pathway to getting the needed help may be self-guided or initiated by a peer, chaplain, or other VA employee. By training an occupationally diverse cadre of VA staff to be capable and willing to share mental health resources with veterans, we can potentially reach a wide range of veterans who can benefit from these tools.

## Abbreviations

AIMS: Anger & Irritability Management Skills
AOSL: all other service lines
DoD: Department of Defense
FE: formative evaluation
MHSL: mental health service line
REDCap: Research Electronic Data Capture
VA MH apps: Veterans Affairs mental health apps
VA: Department of Veterans Affairs
